# Expression levels of the hypothalamic AMPK gene determines the responsiveness of the rats to electroacupuncture-induced analgesia

**DOI:** 10.1186/1472-6882-14-211

**Published:** 2014-06-30

**Authors:** Sun Kwang Kim, Boram Sun, Heera Yoon, Ji Hwan Lee, Giseog Lee, Sung-Hwa Sohn, Hyunseong Kim, Fu Shi Quan, Insop Shim, Joohun Ha, Byung-Il Min, Hyunsu Bae

**Affiliations:** 1 Department of Physiology, College of Korean Medicine, Kyung Hee University, 130-701 Seoul, Republic of Korea; 2 Department of East-West Medicine, Graduate School, Kyung Hee University, 130-701 Seoul, Republic of Korea; 3 Department of Microbiology, Pusan National University, 609-735 Busan, Republic of Korea; 4 Department of Physiology, School of Medicine, Ajou University, 443-721 Suwon, Republic of Korea; 5 Department of Medical Zoology, School of Medicine, Kyung Hee University, 130-701 Seoul, Republic of Korea; 6 Acupuncture & Meridian Science Research Center, Kyung Hee University, 130-701 Seoul, Republic of Korea; 7 Department of Biochemistry and Molecular Biology, School of Medicine, Kyung Hee University, 130-701 Seoul, Republic of Korea; 8 Department of Physiology, School of Medicine, Kyung Hee University, 130-701 Seoul, Republic of Korea

**Keywords:** Electroacupuncture, Analgesia, 5’-AMP-activated protein kinase, Responder, Nonresponder, Hypothalamus, Adenovirus, Rats

## Abstract

**Background:**

Although electroacupuncture (EA) relieves various types of pain, individual differences in the sensitivity to EA analgesia have been reported, causing experimental and clinical difficulties. Our functional genomic study using cDNA microarray identified that 5’-AMP-activated protein kinase (AMPK), a well-known factor in the regulation of energy homeostasis, is the most highly expressed gene in the hypothalamus of the rats that were sensitive to EA analgesia (“responder”), as compared to the rats that were insensitive to EA analgesia (“non-responder”). In this study, we investigated the causal relationship between the hypothalamic AMPK and the individual variation in EA analgesia.

**Methods:**

Sprague-Dawley (SD) rats were divided into the responder and the non-responder groups, based on EA-induced analgesic effects in the tail flick latency (TFL) test, which measures the latency of the tail flick response elicited by radiant heat applied to the tail. Real-time reverse transcription-polymerase chain reaction (RT-PCR) was performed to quantify the expression levels of AMPK mRNA in the hypothalamus of the responder and non-responder rats. Further, we examined whether viral manipulation of the AMPK expression in the hypothalamus modulates EA analgesia in rats.

**Results:**

The real-time RT-PCR analysis showed that mRNA expression levels of AMPK in the hypothalamus of the responder rats are significantly higher than those of the non-responder rats, validating the previous microarray results. Microinjection of dominant negative (DN) AMPK adenovirus, which inhibits AMPK activity, into the rat hypothalamus significantly attenuates EA analgesia (*p* < 0.05), whereas wild type (WT) AMPK virus did not affect EA analgesia (*p* > 0.05).

**Conclusions:**

The present results demonstrated that levels of AMPK gene expression in the rat hypothalamus determine the individual differences in the sensitivity to EA analgesia. Thus, our findings provide a clinically useful evidence for the application of acupuncture or EA for analgesia.

## Background

Acupuncture has been traditionally used for thousands of years in East Asia including China, Korea and Japan to relieve pain and is now viewed as an alternative method of medicine in Western countries [1,2]. Electroacupuncture (EA) is a modified technique that utilizes electrical stimulation to enhance the analgesic effects of acupuncture [3,4]. Previous studies have shown that acupuncture or EA stimulation at specific acupoints (e.g. ST36 and HI4) relieves various types of pain including acute thermal, inflammatory and chronic neuropathic pain, which were known to be mediated by activation of the descending pain inhibitory system [3,5-7]. However, there have been many reports showing individual differences in the sensitivity to EA analgesia, which cause experimental and clinical difficulties: About 30-40% of rats were insensitive to EA in an acute thermal pain test, tail flick latency (TFL) test [5,8,9]. The similar results could be observed in the rat models of inflammatory and neuropathic pain [10,11].

Using cDNA microarray study in the rat hypothalamus, a center of the descending pain inhibitory system, we previously identified several genes that mediate the individual variation in the sensitivity to EA analgesia [12]: The expression levels of 5’-AMP-activated protein kinase (AMPK), dopamine beta-hydroxylase (DBH), acetylcholinesterase T subunit (AChET) in the hypothalamus of the responder rats were significantly higher than those of the non-responder rats. Since cDNA microarray alone could be subject to errors through cross-hybridization, the gene expressions for further study should be validated using new RNA samples [13]. Indeed, our previous study using real-time RT-PCR confirmed that the mRNA expressions of AChET and DBH in the responder group were greater than those in the non-responder one [14]. We also demonstrated that overexpression of AChET [15] or DBH [16] in the rat hypothalamus by viral gene transfer significantly potentiates EA analgesia. However, the post-microarray validation of AMPK and its functional role in EA analgesia have not been studied, despite the highest expression of AMPK in the responder rats as compared to the non-responders among the above three genes.

AMPK has a key role in the regulation of energy balance at both the cellular and whole-body levels, placing it at the center stage in studies of metabolic disorders [17]. Recently, AMPK has also been identified as a potential target for therapy of acute and chronic pain [18,19]. In the present study, we investigated the relationship between the hypothalamic AMPK and the individual variation in EA analgesia by using real-time RT-PCR and genetic manipulation. We report here that the expression levels of AMPK gene in the hypothalamus play an important role in determining the individual differences in the sensitivity to EA analgesia in rats.

## Methods

### Animals

Adult male Sprague-Dawley rats (7 weeks old) (Daehan biolink, Chungbuk, Korea) were housed in cages (3-4 rats per cage) with water and food available ad libitum. The room was maintained with a 12 h-light/dark cycle (a light cycle; 08:00-20:00, a dark cycle; 20:00-08:00) and kept at 23 ± 2°C. All animals were acclimated in their cages for 1 week prior to any experiments. All procedures involving animals were approved by the Institutional Animal Care and Use Committee of Kyung Hee University [KHUASP(SE)-12-013] and were conducted in accordance with the guidelines of the International Association for the Study of Pain [20].

### Acute thermal pain behavior: TFL test

The analgesic effects of EA on acute thermal pain were quantified using the TFL test, which measures the latency of the tail flick response elicited by radiant heat applied to the proximal third of the tail [8,10]. In order to minimize the any possible stress during the TFL testing and EA stimulation, a period of 3 weeks was allowed for adaptation of rats to handling. The rats were individually placed on the palm of an experimenter’s hand and the back was continuously and softly stroked. Then, the rats could be kept calm without the need for anesthetics or holder restrainers [8,21]. For TFL test, the intensity of the light bulb was set such that the baseline reaction time was 3.0 ± 0.5 sec during the pre-test period. In the experimental period, three successive determinations of TFL using the same intensity of the light bulb that had been determined during the pre-test period were conducted at 1-min intervals with a cut-off time of 15 sec, and these values were averaged (pre-EA TFL). For EA stimulation, a pair of stainless steel acupuncture needles (0.25 mm in diameter and 3 cm long) was inserted (5 mm in depth) into the “Zusanli” acupoint (ST36), which is located in the anterior tibial muscle, 5 mm lateral and distal to the anterior tubercle of the tibia, and into the point 5 mm distal from the first needle. EA stimulation at this point is known to produce analgesia in rats [3,8]. An electrical stimulator was connected to the two acupuncture needles (cathode to ST36 and anode to the other point), and train-pulses (2 Hz, 0.5 ms pulse duration, 0.2-0.3 mA) were then applied for 20 minutes. The average of three successive TFL determinations (post-EA TFL) was then recorded. The analgesic effects are expressed as percent changes from the pre-EA TFL.

$$\text{Acquired}\;\text{TFL}\;\text{change}\;\left(\%\right)\;=\frac{\text{Post}-\text{EA}\;\text{TFL}\;–\text{Pre}-\text{EATFL}}{\text{Pre}-\text{EA}\;\text{TFL}}\times \;100$$

The rats showing a TFL increase after EA stimulation that was greater than 30% were classified as responders (mean TFL increase ratio = 59.00%, n = 10), whereas the rats showing less than a 20% TFL increase as non-responders (mean TFL increase ratio = 8.25%, n = 8). Since the other subjects (20-30% TFL increase after EA) are ambiguous for a clear classification, those rats were discarded [15].

### Real-time RT-PCR

Rats in both groups were rapidly sacrificed after EA stimulation and TFL test, and the hypothalamus were separated. RNA was then isolated from the hypothalamus using a Trizol reagent (Invitrogen) according to the manufacturer’s instructions, after which the RNA was quantified using a model ND-1000 apparatus (NanoDrop Technologies, Wilmington, DE, USA). The integrity of the RNA was confirmed by denaturing agarose gel electrophoresis. Single-stranded cDNA was prepared using First Strand cDNA Synthesis Kit (Roche Diagnostics Korea Applied Science, Seoul, Korea). The integrity of the cDNA was confirmed by amplifying GAPDH. The real-time PCR was conducted by a LightCycler 480 (Roche Applied Science, Indianapolis, IN) employing SYBR Green I as the dsDNA-specific binding dye for continuous fluorescence monitoring. The PCR protocol comprised 10 min at 95°C; 45 cycles of 10s at 95°C, 10s at 60°C and 10s at 72°C. After the cycles were finished, the signal of each temperature between 65 and 95°C was also detected to generate a dissociation curve. The sequences of the human primers were AMPK (forward 5’-tgaagccagagaacgtgttg-3’, reverse 5’- ataatttggcgatccacagc-3’) and GAPDH (forward 5’-tgccactcagaagactgtgg-3’, reverse 5’-ttcagctctgggatgacctt-3’). The mRNA levels of AMPK were compared by calculating the crossing point (Cp) value and normalized by the reference genes (GAPDH) using the LightCycler 480 Relative Quantification software (Roche).

### Production of adenovirus vector

AMPK wild type α subunit (WT) and a dominant negative form (DN), in which Asp^157^ was replaced with alanine, were generated by PCR as previously described [22]. The early region 1-deleted recombinant adenoviral vector encoding AMPK α subunit was generated by introducing AMPK cDNA into the shuttle plasmid pAv1 under the transcriptional control of the cytomegalovirus immediately early enhancer/promoter [23]. The recombinant shuttle plasmid was cotransfected with the early region 1-deleted adenovirus serotype 5 genome, pJM17, and amplified in HEK 293 cells. The recombinant adenoviruses were purified by two centrifugation steps on cesium chloride gradients and dialyzed against 10 mM Tris-HCl, pH 8.0, 1 mM MgCl_2_, and 10% glycerol. The number of viral particles was assessed by measurement of the optical density at 260 nm [24]. The titers of GFP control, WT and DN AMPK viruses were 1.5 × 10^12^ pfu/ml, 2.0 × 10^12^ pfu/ml and 2.0 × 10^12^ pfu/ml, respectively.

### Microinjection of adenovirus into the hypothalamus

**Figure 1:**
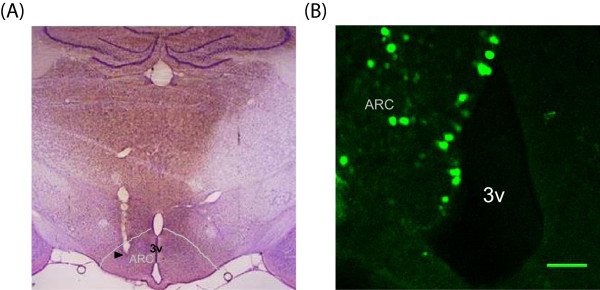
**Verification of the correct injection and transfection of the adenovirus into hypothalamus. (A)** Representative photograph (×40) of the Nissle staining showing the injection position (arrowhead). **(B)** Representative confocal microphotograph of GFP fluorescence in the hypothalamic arcuate nucleus (ARC) from the rat injected with adenovirus. 3v, 3rd ventricle. Scale bar, 100 μm.

Under isoflurane anesthesia, the rat’s head was fixed in a stereotaxic instrument (Stoelting, USA). After a longitudinal incision of the scalp, the skull was drilled to make a hole over the hypothalamic arcuate nucleus (-3.8 anterior-posterior, 0.5 mediolateral, 9.8 dorsoventral, according to the atlas of Paxinos and Watsons [25]). Two microliters of WT or DN AMPK adenoviruses’ viral suspension were injected unilaterally into the hypothalamus at a rate of 0.2 ul/min, using a 10 ul Hamiton syringe (30 gauge beveled needle) attached to a Nano-injector, stepper motorized (Stoleting). The syringe was left in place for 10 min after microinjection and then withdrawn very slowly over 10 min. The skin was sutured with metal wound clips and the rats were allowed to recover from surgery. In a subset of rats, GFP control virus was co-administered with WT or DN AMPK adenovirus to confirm the transfection of viruses and correct injection of adenovirus was verified by Nissle staining (Figure 1).

### Statistical analysis

All the data are presented as mean ± SEM. Statistical analysis was done with Prism 5.0 (Graph Pad Software, USA). The unpaired t-test was used for statistical analysis. In all cases, *p* < 0.05 was considered significant.

## Results

### Measurement of AMPK mRNA levels in the rat hypothalamus by real-time RT-PCR

**Figure 2:**
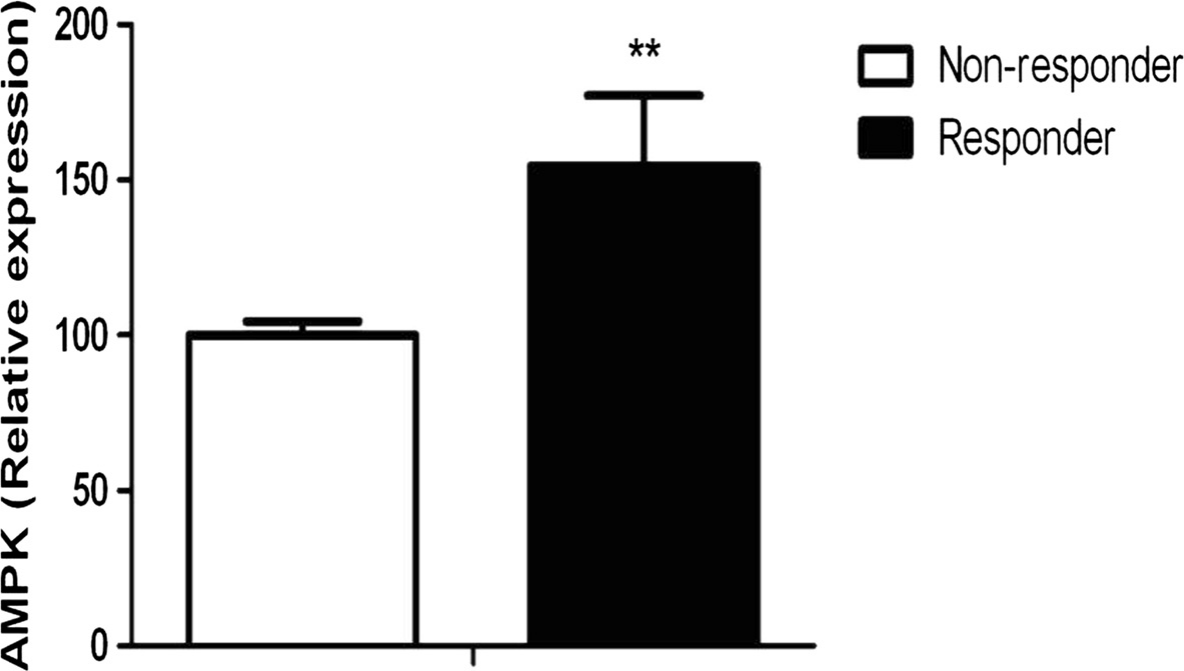
**Normalized mRNA level of the hypothalamic AMPK in the “responder” and “non-responder” rats.** Real-time RT-PCR experiments show the amount of AMPK mRNA expression that normalized by dividing AMPK intensities by that of the house keeping gene, GAPDH. Data are presented as mean ± SEM. **p < 0.01, responder (n = 4) vs. non-responder (n = 4) by the unpaired t-test.

For each group (i.e. responder group and non-responder group), 4 subjects were rapidly sacrificed and the hypothalamus was separated. RNA was extracted from the hypothalamus and the real-time RT-PCR was performed. Expression level of AMPK mRNA was normalized by that of a house keeping gene, GAPDH (Glyceraldehyde-3-phosphate dehydrogenase). As shown in Figure 2, the normalized mRNA levels of AMPK in the responder rats are significantly higher than those of non-responder rats (*p* < 0.01).

### Effects of adenoviral gene transfer of AMPK into the hypothalamus on EA-induced analgesia

**Figure 3:**
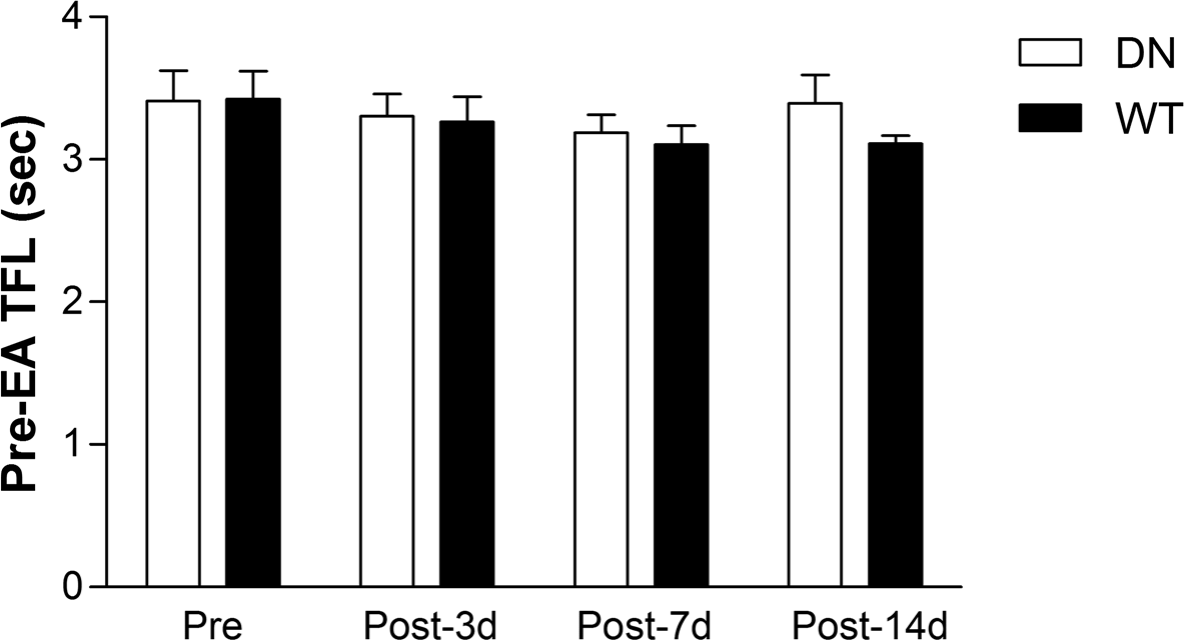
**Time course of pre-EA TFL in WT AMPK and DN AMPK virus-injected rats.** The TFL was measured before EA stimulation on days -1, 3, 7 and 14 following viral injection. No significant differences in pre-EA TFL were observed between the WT virus-injected and DN virus-injected rats during the whole experimental period. Data are presented as mean ± SEM. N = 8/group.

In order to determine whether adenoviral gene transfer of AMPK into the rat hypothalamus by itself affects the sensitivity to thermal stimuli, we compared the baseline TFL between the AMPK WT virus-injected and DN virus-injected rats that measured before EA stimulation on days -1, 3, 7 and 14 following viral injection. There were no significant differences in these pre-EA TFL values between the WT virus-injected and DN virus-injected rats during 2-week experimental period (*p* > 0.05, Figure 3).

**Figure 4:**
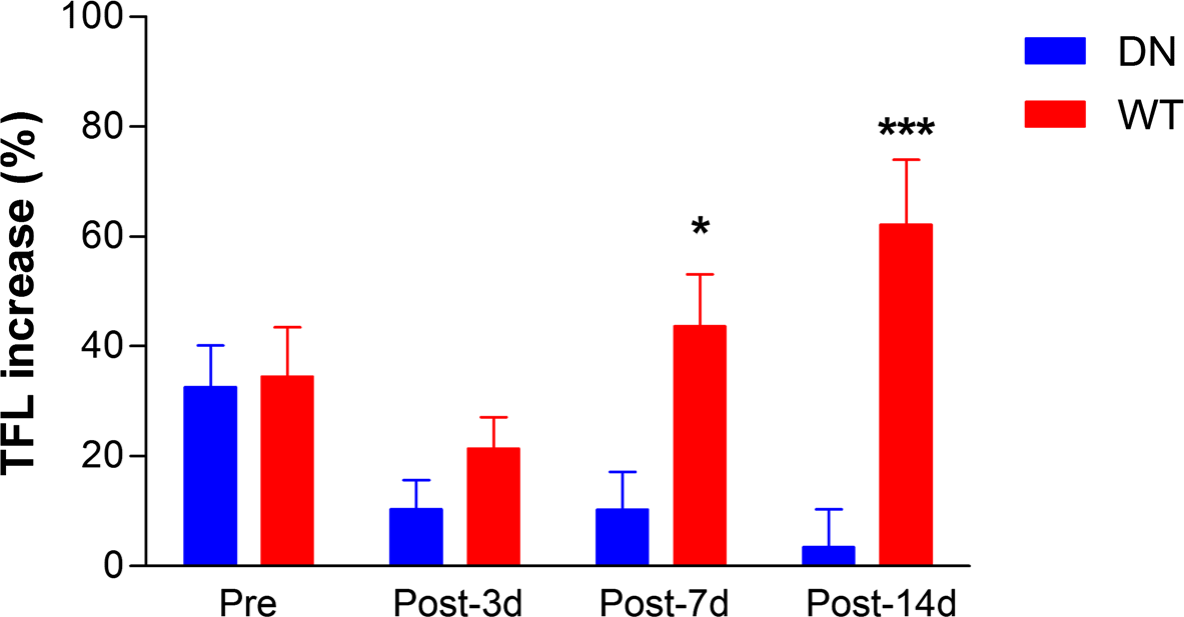
**Comparison of TFL increase ratio after EA between WT AMPK and DN AMPK virus-injected rats.** DN: dominant negative form AMPK virus-injected rats (n = 8); WT: wild-type AMPK α subunit virus-injected rats (n = 8). Pre: before the microinjection of virus; Post: after virus microinjection. Data are presented as mean ± SEM. *p < 0.05 and ***p < 0.001, WT vs. DN by the unpaired *t*-test.

To see whether WT AMPK virus and DN AMPK virus gene expression in the hypothalamus alter EA-induced analgesic effects, we compared the TFL increase ratio between the WT AMPK virus-injected and DN AMPK virus-injected rats. In consistent with the role of DN AMPK virus transfection in inhibiting AMPK activity [24,26], EA-induced analgesic effects were markedly decreased in a time dependent manner after microinjection of DN AMPK virus into the hypothalamus (Figure 4). DN AMPK virus-injected rats showed a significant decrease in TFL increase ratio after EA at 14 days post-injection as compared to the value at pre-injection day (*p* < 0.05). Conversely, WT AMPK virus-injected rats showed no significant difference in TFL increase ratio between the pre-injection day and the post-injection days (*p* > 0.05). Comparison of the TFL increase ratio shows a significant difference between the WT and DN AMPK virus-injected rats on the 7th (*p* < 0.05) and 14th (*p* < 0.001) days following the injection (Figure 4).

## Discussion

Pain is considered both a sensation and an emotion, showing considerable complexity and subjectivity. In clinical and laboratory settings, the perception of pain bears a poor relationship to the intensity of the noxious stimulus [27]. Therefore, strong interest exists in understanding the individual differences in response to pain and analgesics. To elucidate the genetic contributions to such individual variability in animals and humans, researchers are now employing a variety of approaches, such as microarray analysis, epigenetics and human brain imaging [13,28,29].

The analgesic effects of EA also show marked individual differences in acute, inflammatory and neuropathic pain rats [5,8-11]. To identify and characterize the genes that cause these individual differences in response to EA analgesia, we previously conducted cDNA microarray analysis, using the hypothalamus, a main center of EA analgesia and the descending pain inhibitory system [12]. Among several genes that are more abundantly expressed in the responder rats than non-responder rats, AMPK gene is the most differently expressed between the two groups. In the present study, we confirmed this with a real-time RT-PCR (Fiure 3) strongly suggesting that the expression of AMPK in the hypothalamus is closely associated with individual differences in response to EA analgesia. This study further validated the results by using viral gene transfer of AMPK into the hypothalamus (Figure 4). EA-induced analgesic effects were gradually decreased and slightly increased after injection of DN AMPK virus and WT AMPK virus, respectively, producing a significant difference between the two groups at 7 and 14 days post-injection.

The mammalian AMPK is a heterotrimer consisting of an α catalytic subunit and β and γ noncatalytic subunit [26]. Isolation of AMPK to homogenously revealed that the catalytic subunit (α) co-purifies with two other noncatalytic subunit (β and γ). The formation of a trimeric subunit complex is necessary for an optimal AMPK activity and it is known that overexpression of wild type α subunit does not exert any positive effect on an endogenous AMPK activity [24,26]. Consistent with these reports, there was no significant increase in EA-induced analgesic effect after WT AMPK virus injection. Conversely, the inhibition of AMPK activity by DN AMPK virus injection significantly decreased the EA analgesia (Figure 4).

AMPK is primarily regulated by cellular AMP/ATP and nutrient levels and plays a central role in the regulation of energy homeostasis and metabolic stress [30]. It has emerged as a promising new drug target for treatment metabolic disorders, including obesity, type 2 diabetes and cardiovascular disease [17]. Several studies also suggested that AMPK activation plays a significant role in important neuronal processes, including the regulation of neuronal plasticity and long-term potentiation, and the protection of neurons from neurodegenerative diseases [31]. Although there has been little research on the role of AMPK in nociception, very recent studies demonstrated that AMPK activation significantly alleviates acute, inflammatory and neuropathic pain through the modulation of mammalian target of rapamycin (mTOR) and mitogen activated protein kinase (MAPK) signaling in the periphery and spinal cord that are related to pain hypersensitivity [18,19,32]. Our data further demonstrated that the hypothalamic AMPK play a role in mediating individual differences in response to EA-mediated analgesia. Thus, these findings not only provide a clinically useful evidence for the application of acupuncture or EA for analgesia, but also suggest an unexpected role of the hypothalamic AMPK in pain modulation.

It is currently unclear how the hypothalamic AMPK plays a role in EA-induced analgesia as shown in this study. One possible explanation is that AMPK might regulate EA analgesia-related neuropeptides that released in the hypothalamus. AMPK activation in the hypothalamus is positively correlated with neuropeptide Y (NPY) expressions [33] and this hypothalamic NPY has a significant antinociceptive effect [34]. Interestingly, several reports demonstrated that acupuncture or EA stimulation at ST36 decreases NPY levels in the hypothalamus [35,36]. Thus, we cautiously assumed that the responder rats with high AMPK levels, but not non-responders, might maintain sufficient NPY levels in the hypothalamus to be involved in antinociception, although EA stimulation decreased NPY expressions. In addition to this, further studies to explore the relationship between the AMPK and beta-endorphin in the hypothalamus, a well-known EA analgesia mediator, are required. Also, it would be interesting to examine the analgesic effects of EA on pathological pain, such as neuropathic pain [37], the mechanism of which is somewhat different from acute pain (e.g. TFL test). Although the individual differences in the sensitivity of acute nociceptive and chronic neuropathic pain to EA in rats were known to be maintained [10], we believe that studies using pathological pain models could provide a better understanding of EA-induced analgesia and its responsiveness.

## Conclusions

In conclusion, we demonstrate that mRNA expression of AMPK in the hypothalamus of the responder rats is significantly higher than the non-responder rats. Furthermore, adenoviral gene transfer of AMPK in the hypothalamus could alter the EA-induced analgesia. Taken together, these results strongly suggest that levels of AMPK gene expression in the rat hypothalamus determine the individual differences in the sensitivity to EA analgesia.

## Competing interests

The authors declare that they have no competing interests.

## Authors’ contributions

SKK, BIM and HB contributed to the conception and design of the study. SKK, BS, HY, JHL, GL, HK, FSQ and IS performed the experiments and analyzed the data. JH provided the DN and WT AMPK viruses. SKK, BS and HB wrote the manuscript. All authors read and approved the final manuscript.

## Pre-publication history

The pre-publication history for this paper can be accessed here:

http://www.biomedcentral.com/1472-6882/14/211/prepub

## References

[B1] Cherkin DC Sherman KJ Deyo RA Shekelle PG A review of the evidence for the effectiveness, safety, and cost of acupuncture, massage therapy, and spinal manipulation for back pain Ann Intern Med 2003 138 11 898 906 12779300 10.7326/0003-4819-138-11-200306030-00011

[B2] Kaptchuk TJ Acupuncture: theory, efficacy, and practice Ann Intern Med 2002 136 5 374 383 11874310 10.7326/0003-4819-136-5-200203050-00010

[B3] Kim SK Park JH Bae SJ Kim JH Hwang BG Min BI Park DS Na HS Effects of electroacupuncture on cold allodynia in a rat model of neuropathic pain: mediation by spinal adrenergic and serotonergic receptors Exp Neurol 2005 195 2 430 436 16054138 10.1016/j.expneurol.2005.06.018

[B4] Schliessbach J van der Klift E Arendt-Nielsen L Curatolo M Streitberger K The effect of brief electrical and manual acupuncture stimulation on mechanical experimental pain Pain Med 2011 12 2 268 275 21276188 10.1111/j.1526-4637.2010.01051.x

[B5] Han J The neurochemical basis of pain relief by acupuncture 1987 Beijing: Chinese Medical Science and Technology Press

[B6] Takeshige C Sato T Mera T Hisamitsu T Fang J Descending pain inhibitory system involved in acupuncture analgesia Brain Res Bull 1992 29 5 617 634 1422859 10.1016/0361-9230(92)90131-g

[B7] Zhang RX Lao L Wang L Liu B Wang X Ren K Berman BM Involvement of opioid receptors in electroacupuncture-produced anti-hyperalgesia in rats with peripheral inflammation Brain Res 2004 1020 1–2 12 17 15312782 10.1016/j.brainres.2004.05.067

[B8] Lee G Rho S Shin M Hong M Min B Bae H The association of cholecystokinin-A receptor expression with the responsiveness of electroacupuncture analgesic effects in rat Neurosci Lett 2002 325 1 17 20 12023057 10.1016/s0304-3940(02)00214-8

[B9] Takeshige C Murai M Tanaka M Hachisu M Parallel individual variations in effectiveness of acupuncture, morphine analgesia, and dorsal PAG-SPA and their abolition by D-phenylalanine Adv Pain Res Ther 1983 5 563 569

[B10] Kim SK Moon HJ Park JH Lee G Shin MK Hong MC Bae H Jin YH Min BI The maintenance of individual differences in the sensitivity of acute and neuropathic pain behaviors to electroacupuncture in rats Brain Res Bull 2007 74 5 357 360 17845910 10.1016/j.brainresbull.2007.07.006

[B11] Sekido R Ishimaru K Sakita M Differences of electroacupuncture-induced analgesic effect in normal and inflammatory conditions in rats Am J Chin Med 2003 31 6 955 965 14992547 10.1142/S0192415X03001491

[B12] Lee G Rho S Lee J Min BI Hong M Bae H Cloning of genes responsible for distinguishing between responder and non-responder to the acupuncture mediated analgesic effects Experimental Biology 2001 2001 Orlando: The FASEB journal 1166

[B13] Costigan M Griffin RS Woolf C Mogil JS Microarray analysis of the pain pathway The Genetics of Pain 2004 Seattle: IASP press 65 84

[B14] Sur Y Rho S Lee G Ko E Hong M Shin M Min B Bae H Gene expression profile of the responder vs. the non-responder to the acupuncture mediated analgesic effects Korean J Orient Physiol Pathol 2003 17 633 642

[B15] Kim SK Park JY Koo BH Lee JH Kim HS Choi WK Shim I Lee H Hong MC Shin MK Min BI Bae H Adenoviral gene transfer of acetylcholinesterase T subunit in the hypothalamus potentiates electroacupuncture analgesia in rats Genes Brain Behav 2009 8 2 174 180 19077179 10.1111/j.1601-183X.2008.00459.x

[B16] Kim SJ Chung ES Lee JH Lee CH Kim SK Lee HJ Bae H Electroacupuncture analgesia is improved by adenoviral gene transfer of dopamine beta-hydroxylase into the hypothalamus of rats Korean J Physiol Pharmacol 2013 17 6 505 510 24381499 10.4196/kjpp.2013.17.6.505 PMC3874437

[B17] Yun H Ha J AMP-activated protein kinase modulators: a patent review (2006–2010) Expert Opin Ther Pat 2011 21 7 983 1005 21548715 10.1517/13543776.2011.577069

[B18] Melemedjian OK Asiedu MN Tillu DV Sanoja R Yan J Lark A Khoutorsky A Johnson J Peebles KA Lepow T Sonenberg N Dussor G Price TJ Targeting adenosine monophosphate-activated protein kinase (AMPK) in preclinical models reveals a potential mechanism for the treatment of neuropathic pain Mol Pain 2011 7 70 21936900 10.1186/1744-8069-7-70 PMC3186752

[B19] Tillu DV Melemedjian OK Asiedu MN Qu N De Felice M Dussor G Price TJ Resveratrol engages AMPK to attenuate ERK and mTOR signaling in sensory neurons and inhibits incision-induced acute and chronic pain Mol Pain 2012 8 5 22269797 10.1186/1744-8069-8-5 PMC3284441

[B20] Zimmermann M Ethical guidelines for investigations of experimental pain in conscious animals Pain 1983 16 2 109 110 6877845 10.1016/0304-3959(83)90201-4

[B21] Ko ES Kim SK Kim JT Lee G Han JB Rho SW Hong MC Bae H Min BI The difference in mRNA expressions of hypothalamic CCK and CCK-A and -B receptors between responder and non-responder rats to high frequency electroacupuncture analgesia Peptides 2006 27 7 1841 1845 16472889 10.1016/j.peptides.2006.01.002

[B22] Woods A Azzout-Marniche D Foretz M Stein SC Lemarchand P Ferre P Foufelle F Carling D Characterization of the role of AMP-activated protein kinase in the regulation of glucose-activated gene expression using constitutively active and dominant negative forms of the kinase Mol Cell Biol 2000 20 18 6704 6711 10958668 10.1128/mcb.20.18.6704-6711.2000 PMC86183

[B23] Kobayashi K Oka K Forte T Ishida B Teng B Ishimura-Oka K Nakamuta M Chan L Reversal of hypercholesterolemia in low density lipoprotein receptor knockout mice by adenovirus-mediated gene transfer of the very low density lipoprotein receptor J Biol Chem 1996 271 12 6852 6860 8636110 10.1074/jbc.271.12.6852

[B24] Lee M Hwang JT Lee HJ Jung SN Kang I Chi SG Kim SS Ha J AMP-activated protein kinase activity is critical for hypoxia-inducible factor-1 transcriptional activity and its target gene expression under hypoxic conditions in DU145 cells J Biol Chem 2003 278 41 39653 39661 12900407 10.1074/jbc.M306104200

[B25] Paxinos G Watson C The rat brain in stereotaxic coordinates 1998 San Diego: Academic 10.1016/0165-0270(80)90021-7 6110810

[B26] Dyck JR Gao G Widmer J Stapleton D Fernandez CS Kemp BE Witters LA Regulation of 5'-AMP-activated protein kinase activity by the noncatalytic beta and gamma subunits J Biol Chem 1996 271 30 17798 17803 8663446 10.1074/jbc.271.30.17798

[B27] Mogil JS The genetic mediation of individual differences in sensitivity to pain and its inhibition Proc Natl Acad Sci U S A 1999 96 14 7744 7751 10393892 10.1073/pnas.96.14.7744 PMC33613

[B28] Crow M Denk F McMahon SB Genes and epigenetic processes as prospective pain targets Genome Med 2013 5 2 12 23409739 10.1186/gm416 PMC3706821

[B29] Zubieta JK Heitzeg MM Smith YR Bueller JA Xu K Xu Y Koeppe RA Stohler CS Goldman D COMT val158met genotype affects mu-opioid neurotransmitter responses to a pain stressor Science 2003 299 5610 1240 1243 12595695 10.1126/science.1078546

[B30] Ramamurthy S Ronnett GV Developing a head for energy sensing: AMP-activated protein kinase as a multifunctional metabolic sensor in the brain J Physiol 2006 574 Pt 1 85 93 16690704 10.1113/jphysiol.2006.110122 PMC1817796

[B31] Price TJ Dussor G AMPK: an emerging target for modification of injury-induced pain plasticity Neurosci Lett 2013 557 Pt A 9 18 23831352 10.1016/j.neulet.2013.06.060 PMC3844111

[B32] Russe OQ Moser CV Kynast KL King TS Stephan H Geisslinger G Niederberger E Activation of the AMP-activated protein kinase reduces inflammatory nociception J Pain 2013 14 11 1330 1340 23916727 10.1016/j.jpain.2013.05.012

[B33] Stark R Ashley SE Andrews ZB AMPK and the neuroendocrine regulation of appetite and energy expenditure Mol Cell Endocrinol 2013 366 2 215 223 22789749 10.1016/j.mce.2012.06.012

[B34] Li JJ Zhou X Yu LC Involvement of neuropeptide Y and Y1 receptor in antinociception in the arcuate nucleus of hypothalamus, an immunohistochemical and pharmacological study in intact rats and rats with inflammation Pain 2005 118 1–2 232 242 16216414 10.1016/j.pain.2005.08.023

[B35] Eshkevari L Egan R Phillips D Tilan J Carney E Azzam N Amri H Mulroney SE Acupuncture at ST36 prevents chronic stress-induced increases in neuropeptide Y in rat Exp Biol Med 2012 237 1 18 23 10.1258/ebm.2011.011224 22156045

[B36] Lee JD Jang MH Kim EH Kim CJ Acupuncture decreases neuropeptide Y expression in the hypothalamus of rats with Streptozotocin-induced diabetes Acupunct Electrother Res 2004 29 1–2 73 82 15382790 10.3727/036012904815901533

[B37] Kim W Kim SK Min BI Mechanisms of electroacupuncture-induced analgesia on neuropathic pain in animal model eCAM 2013 2013 436913 23983779 10.1155/2013/436913 PMC3747484

